# Doping Efficiency of Poly(benzodifurandione) from First Principles

**DOI:** 10.1021/acs.jpcc.4c07765

**Published:** 2025-02-21

**Authors:** Paolo S. Floris, Igor Zozoulenko, Riccardo Rurali

**Affiliations:** † Institut de Ciència de Materials de Barcelona, ICMAB-CSIC, Campus UAB, 08193 Bellaterra, Spain; ‡ Laboratory of Organic Electronics (LOE), Department of Science and Technology (ITN), Linköping University, Campus Norrköping, 60174 Norrköping, Sweden

## Abstract

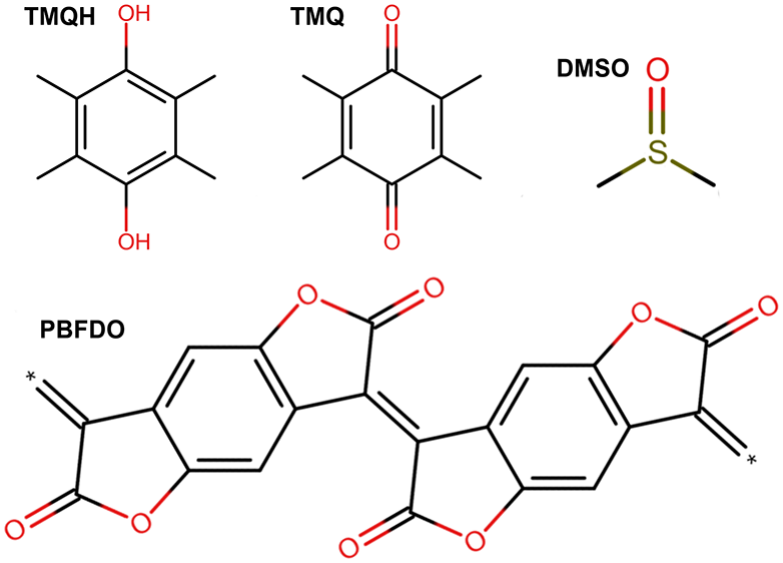

Poly(benzodifurandione) (PBFDO) has emerged as a promising n-type conductive polymer (n-CP) for organic electronic applications, particularly in thermoelectrics (TE), due to its high doping efficiency and environmental stability. Unlike most high-performance p-type polymers, high-efficiency n-CPs are limited, posing a bottleneck in the TE module performance. In this study, we use first-principles electronic structure calculations to investigate the thermodynamic conditions that favor n-doping in PBFDO, focusing on the role of the temperature, chain length, and doping concentration. We compute the change in Gibbs free energy, Δ*G*, upon doping and explore how it varies with temperature and polymer chain length. Our results show that doping becomes more thermodynamically favorable at lower temperatures and in longer chains, with a strong dependence of Δ*G* on the doping level emerging as chain length increases. Notably, PBFDO can achieve favorable doping levels across various chain lengths and temperatures, with specific doping thresholds identified for different molecular weights. These findings suggest that lower synthesis temperatures could lead to more heavily doped, higher-conductivity PBFDO, and that chain length significantly influences achievable doping efficiency. This work provides insights for optimizing PBFDO doping strategies to enhance its performance in TE applications, bridging a key gap in organic semiconductor research.

## Introduction

Poly(benzodifurandione) (PBFDO) is a solution-processed, sidechain-free n-type conductive polymer (n-CP)^1^ that has recently attracted significant attention in fields such as organic electronics^2−5^ and, in particular, thermoelectrics (TE).^6−8^ The main reason for this widespread interest is that, while a number of high-performance p-type CPs (p-CPs) are already available,^9−11^ n-CPs with such remarkable performances (in terms of both doping efficiency and environmental stability) have proven extremely difficult to produce. In their most usual configuration, TE modules consist of an n- and a p-type leg that are thermally connected in parallel and electrically in series. Therefore, the poor performances of n-CPs constitute an important efficiency bottleneck for these devices and is one of the most pressing issues in the research agenda of organic semiconductors.

PBFDO is synthesized by means of oxidative polymerization and n-doped in situ, leading to a fairly high doping efficiency (86%). It exhibits ultrahigh electrical conductivity at room temperature (2000 S cm^–1^) and a good Seebeck coefficient (−21 μV K^–1^), resulting in a power factor of almost 90 W m^–1^ K^–1^.^1^ These figures are lower but comparable to those of the best p-CPs^9−11^ and are among the best-performing n-CPs for TE applications.^12−14^

Despite these exciting results, the scarcity of experimental results to date suggests that the doping efficiency in PBFDO could be improved. To this end, however, a detailed understanding of the interplay between doping and morphology and of the role of temperature is needed and is a prerequisite for any further optimization.

In this work, we present first-principles electronic structure calculations, determining the conditions under which the reaction leading to n-type doped PBFDO is most favorable. In particular, we compute the change in Gibbs free energy upon doping and study its dependence on the temperature and on the polymer chain length, similarly to what has been previously done for poly(3,4-ethylenedioxythiophene) (PEDOT).^15,16^

## Methods

All DFT calculations were carried out with Gaussian 16^18,19^ using the 6-311+G(d,p) basis set.^20,21^ Geometrical optimizations are performed with the hybrid exchange-correlation functional B3LYP^22^ and Grimme’s D3 dispersion correction with Becke-Johnson damping function (GD3BJ).^23,24^ Free energy calculations employ long-range corrected hybrid density functional ωB97X-D.^25^ Furthermore, Gibbs free energies involved in the solvation process are calculated using the Solvation Model based on Density (SMD),^26^ considering DMSO as the implicit solvent (except protons, which are in the gas phase). All other Gibbs free energies are obtained by using the Polarizable Continuum Model (PCM),^27^ with DMSO as the implicit solvent, as well. The initial geometries needed for the calculation of the proton solvation Gibbs free energy were generated with Packmol.^28,29^ We treat isolated molecular systems without periodic boundary conditions.

## Results and Discussion

**Figure 1:**
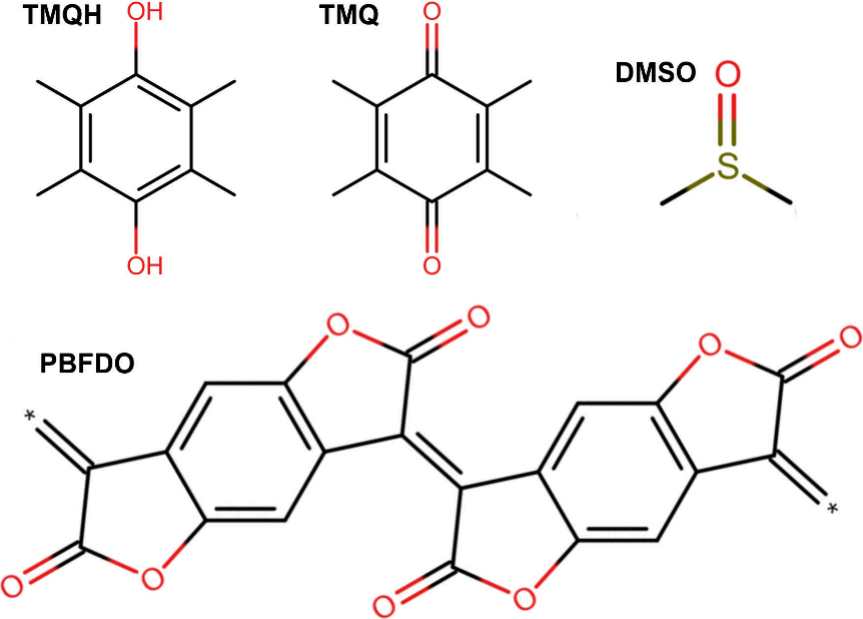
TMQH, TMQ, DMSO, and PBFDO molecular structures. The illustration shows only two monomers. The * symbols indicate the edges of the repeat pattern.

PBFDO (see Figure 1 for a sketch of this and all other molecules discussed in this study) is doped by adding durohydroquinone (TMQH, Figure 1) in a dimethyl sulfoxide (DMSO, Figure 1) solution at *T* = 373 K according to the following reaction:
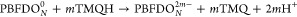
1where TMQ is duroquinone (Figure 1), *N* is the number of monomers that make up the PBFDO polymer chain, and *m* is the number of dopant molecules. However, this reaction does not take into account the solvation of protons within DMSO. This limitation is circumvented by means of the Cluster Continuum Model^17^ (CCM), which uses the following scheme for proton solvation:
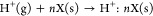
2where *n* is the number of solvent molecules that are explicitly considered, *X* is the solvent, while *s* and *g* refer to solution and gas phase, respectively. Therefore, the complete reaction should be written as
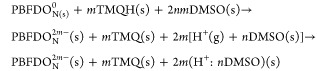
3where the intermediate product is included for clarity, to show exactly where the proton solvation is taking place. The variation of the Gibbs free energy Δ*G* of the doping reaction is simply defined as
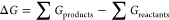
4where the products are on the right-hand side of eq 3, while the reactants are on its left-hand side. The Gibbs free energy variation associated with the proton solvation Δ*G*_solv_ can also be defined, according to eq 2, as
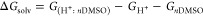
5Using eq 5, Δ*G* can be reformulated in terms of Δ*G*_solv_ as
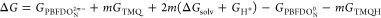
6

PBFDO chains consisting of 4, 6, and 8 monomers were considered (labeled PBFDO_*i*_ hereafter, where *i* represents the number of monomers). Due to the nature of the doping reaction for PBFDO, electrons can only be donated to the polymer in multiples of 2. Therefore, the doping concentrations that can hereby be considered are (50%, 100%) for PBFDO_4_, (33.3%, 66.7%, 100%) for PBFDO_6_ and (25%, 50%, 75%, 100%) for PBFDO_8_, where the doping level *c* is represented as the percent ratio of electrons per monomer. Charge carriers are considered to form uncoupled polarons in all PBFDO samples.

**Figure 2:**
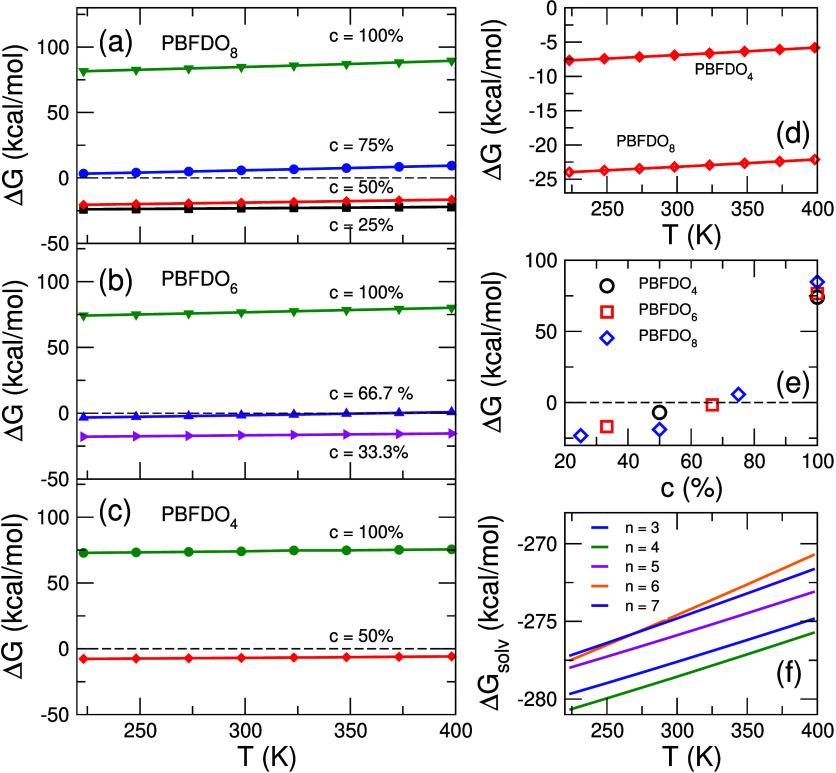
Variation of the Gibbs free energy, Δ*G*, as a function of temperature for (a) PBFDO_8_, (b) PBFDO_6_, and (c) PBFDO_4_ for different levels of doping, *c*. (d) Comparison of Δ*G*(*T*) for *c* = 50% in PBFDO_4_ and PBFDO_8_. (e) Δ*G* at *T* = 298 K for different concentration and chain lengths. (f) Δ*G*_solv_ within the CCM approach for different values of *n* (see text).

The Δ*G*_solv_ of a proton in DMSO, within the CCM approach, is depicted in Figure 2f as a function of *T* and for *n* = 1, ..., 7 (numerical values can be found in the Supporting Information). According to previously published research,^30^*n* ≥ 5 is considered sufficient to obtain realistic results for proton solvation in ammonia, although larger *n* ensures higher accuracy. Since DMSO is a much larger molecule than ammonia, it is expected that it would form a thicker solvation shell, given the same value of *n*. Nonetheless, it was deemed a good compromise between accuracy and computational cost to reach *n* = 7, which allows to achieve convergence (see Figure 2f) in the temperature range considered in this study. The dependence on *T* shows that proton solvation is favored at lower temperatures, a tendency that is in agreement with the literature.^31^

The Δ*G* of the doping reaction is shown in Figure 2a–c for all chain lengths and doping levels, as a function of *T* (numerical values can be found in the Supporting Information). Δ*G* decreases (i.e., the reaction becomes more favorable) in all cases at lower *T*, as exemplified in Figure 2d for *c* = 50%. This tendency can be fully explained by the Δ*G*_solv_ and its dependence on *T*, which is dominant in the calculation of Δ*G* and, thus, determines this behavior (the reader can use the data in the Supporting Information to verify that such a dependence on the temperature would not be observed without taking the proton solvation into account). PBFDO_4_ is favorably doped at *c* = 50% in the entire range of temperatures considered. A doping level *c* = 33.3% is always favorable for PBFDO_6_, while at *c* = 66.7% the reaction only becomes favorable for *T* < 350*K*. Additionally, *c* ≤ 50% is favorable for PBFDO_8_ throughout the entire range of temperatures considered. The results shown in Figure 2e show that for all chain lengths, Δ*G* increases gradually as the doping level ranges from 25 to 75%. However, at *c* = 100%, Δ*G* exhibits a pronounced increase, indicating a strong, abrupt change. This pattern suggests that longer chains may enhance the proportionality between Δ*G* and the doping level. Consequently, the “zero-crossing” of Δ*G* for longer chains could be expected to occur at increasingly higher doping levels. This behavior might explain why Tang et al.^1^ obtained a high doping efficiency of 86% at 373 K, given that their system is composed of very long chains as the solution containing the doped polymer was filtered to remove low molecular weight fractions (below 10 kDa, which corresponds to chains shorter than roughly 54 monomers).

## Conclusions

The findings of the present work indicate that the impact of temperature synthesis on the doping level should be experimentally tested, as lower temperatures could produce even more heavily doped (i.e., more conductive) samples. Likewise, a clear correlation between chain length and achievable doping concentration has emerged, indicating that high doping levels are more easily obtained in high molecular weight PBFDO chains.

## Supporting Information Available

Numerical values of the computed Gibbs free energy (PDF)
