# Supplementary material for: Doping Efficiency of Poly(benzodifurandione) from First Principles
Source: J Phys Chem C Nanomater Interfaces. 2025 Feb 21;129(9):4354–7. doi: 10.1021/acs.jpcc.4c07765 (PMC11891886; doi:10.1021/acs.jpcc.4c07765)
Supplement: Supplementary file 1 — jp4c07765_si_001.pdf [file jp4c07765_si_001.pdf]

Supporting Information for "Doping Efficiency of Poly(benzodifurandione) from First-Principles"

Paolo S. Floris [1], *Igor Zozoulenko* [2], *Riccardo Rurali* [1]

[1]Institut de Ciència de Materials de Barcelona, ICMAB-CSIC, Campus UAB, 08193 Bellaterra, Spain

[2]Laboratory of Organic Electronics (LOE), Department of Science and Technology (ITN), *Linköping University, Campus Norrköping, 60174 Norrköping, Sweden*

E-mail: igor.zozoulenko@liu.se, rrurali@icmab.es

1. Proton Solvation in DMSO

Table 1.1 – Variations of the Gibbs free energy of proton solvation in DMSO. Each column corresponds to a different number of molecules that were explicitly included in the calculation.

Temp (K)	ΔG (kcal/mol)						
	n = 1	n = 2	n = 3	n = 4	n = 5	n = 6	n = 7
223.15	-235.945	-234.740	-279.679	-280.686	-277.989	-277.530	-277.213
248.15	-235.342	-234.126	-279.016	-280.006	-277.319	-276.589	-276.443
273.15	-234.729	-233.501	-278.340	-279.312	-276.637	-275.633	-275.660
298.15	-234.104	-232.864	-277.653	-278.609	-275.943	-274.664	-274.868
323.15	-233.472	-232.218	-276.955	-277.896	-275.240	-273.684	-274.068
348.15	-232.829	-231.564	-276.249	-277.174	-274.528	-272.694	-273.258
373.15	-232.181	-230.901	-275.535	-276.445	-273.808	-271.696	-272.442
398.15	-231.524	-230.233	-274.813	-275.708	-273.081	-270.688	-271.618

Table 1.2 – Absolute Gibbs free energies of a proton in vacuum and of DMSO, with and without including a solvated proton (corresponding to n=7 in Table 1.1).

Temp (K)	G (kcal/mol)		
	H^+	$DMSO_7$	$DMSO_7$:H^+
223.15	-4.375	-2429708.781	-2429990.372
248.15	-4.996	-2429714.977	-2429996.419
273.15	-5.630	-2429721.547	-2430002.840
298.15	-6.275	-2429728.474	-2430009.621
323.15	-6.930	-2429735.748	-2430016.748

348.15	-7.595	-2429743.358	-2430024.214
373.15	-8.269	-2429751.293	-2430032.007
398.15	-8.951	-2429759.547	-2430040.119

2. TMQH and TMQ

Table 2.1 - Absolute Gibbs free energies of TMQH and TMQ.

Temp (K)	G (kcal/mol)	
	**TMQH**	**TMQ**
223.15	-338694.237	-337936.601
248.15	-338696.795	-337939.169
273.15	-338699.467	-337941.839
298.15	-338702.252	-337944.610
323.15	-338705.146	-337947.479
348.15	-338708.148	-337950.445
373.15	-338711.255	-337953.506
398.15	-338714.466	-337956.660

3. $PBFDO_4$

Table 3.1 - Variations of the Gibbs free energy of a $PBFDO_4$ chain.

Temp (K)	ΔG (kcal/mol)	
	**c = 50%**	**c = 100%**
223.15	-7.674	72.889
248.15	-7.425	73.246
273.15	-7.169	73.615
298.15	-6.908	73.990
323.15	-6.641	74.700
348.15	-6.369	74.766
373.15	-6.092	75.166
398.15	-5.812	75.575

Table 3.2 - Absolute Gibbs free energies of a $PBFDO_4$ chain.

Temp (K)	G (kcal/mol)		
	**c = 0%**	**c = 50%**	**c = 100%**
223.15	-1715957.947	-1716160.075	-1716273.964

248.15	-1715962.992	-1716165.160	-1716279.230
273.15	-1715968.377	-1716170.588	-1716284.846
298.15	-1715974.099	-1716176.356	-1716290.809
323.15	-1715980.156	-1716182.463	-1716296.786
348.15	-1715986.544	-1716188.904	-1716303.758
373.15	-1715993.262	-1716195.676	-1716310.738
398.15	-1716000.303	-1716202.775	-1716318.047

4. $PBFDO_6$

Table 4.1 - Variations of the Gibbs free energy of a $PBFDO_6$ chain.

Temp (K)	ΔG (kcal/mol)		
	c = 33.3%	c = 66.7%	c = 100%
223.15	-17.846	-3.296	74.171
248.15	-17.519	-2.747	74.963
273.15	-17.183	-2.182	75.777
298.15	-16.841	-1.605	76.604
323.15	-16.493	-1.015	77.447
348.15	-16.136	-0.409	78.310
373.15	-15.775	0.204	79.182
398.15	-15.408	0.831	80.071

Table 4.2 - Absolute Gibbs free energies of a $PBFDO_6$ chain.

Temp (K)	G (kcal/mol)			
	c = 0%	c = 33.3%	c = 66.7%	c = 100%
223.15	-2573178.844	-2573391.143	-2573571.047	-2573688.032
248.15	-2573185.754	-2573398.016	-2573577.986	-2573695.018
273.15	-2573193.171	-2573405.397	-2573585.438	-2573702.521
298.15	-2573201.093	-2573413.284	-2573593.397	-2573710.537
323.15	-2573209.514	-2573421.673	-2573601.860	-2573719.063
348.15	-2573218.432	-2573430.558	-2573610.822	-2573728.093
373.15	-2573227.839	-2573439.936	-2573620.278	-2573737.620
398.15	-2573237.730	-2573449.798	-2573630.220	-2573747.639

5. $PBFDO_8$

Table 5.1 - Variations of the Gibbs free energy of a $PBFDO_8$ chain.

Temp (K)	ΔG (kcal/mol)			
	c = 25%	c = 50%	c = 75%	c = 100%
223.15	-23.966	-20.481	3.263	81.558
248.15	-23.720	-19.955	4.081	82.593
273.15	-23.465	-19.414	4.926	83.659
298.15	-23.207	-18.864	5.787	84.743
323.15	-22.944	-18.306	6.665	85.848
348.15	-22.675	-17.734	7.566	86.978
373.15	-22.404	-17.159	8.478	88.307
398.15	-22.129	-16.574	9.408	89.484

Table 5.2 - Absolute Gibbs free energies of a $PBFDO_8$ chain.

Temp (K)	G (kcal/mol)				
	c = 0%	c = 25%	c = 50%	c = 75%	c = 100%
223.15	-3430399.099	-3430617.518	-3430808.486	-3430979.195	-3431095.353
248.15	-3430407.817	-3430626.279	-3430817.257	-3430987.963	-3431104.193
273.15	-3430417.212	-3430635.719	-3430826.711	-3430997.413	-3431113.722
298.15	-3430427.277	-3430645.834	-3430836.841	-3431007.540	-3431123.933
323.15	-3430438.007	-3430656.617	-3430847.644	-3431018.339	-3431134.821
348.15	-3430449.396	-3430668.062	-3430859.112	-3431029.802	-3431146.380
373.15	-3430461.436	-3430680.162	-3430871.238	-3431041.922	-3431158.413
398.15	-3430474.119	-3430692.908	-3430884.013	-3431054.691	-3431171.274
